# The Impact of Atmospheric Cadmium Exposure on Colon Cancer and the Invasiveness of Intestinal Stents in the Cancerous Colon

**DOI:** 10.3390/toxics12030215

**Published:** 2024-03-14

**Authors:** Shuai Zhang, Ruikang Li, Jing Xu, Yan Liu, Yanjie Zhang

**Affiliations:** 1 Department of General Surgery, Tianjin Union Medical Center, No. 190 Jieyuan Road, Hongqiao District, Tianjin 300121, China; gelinbarlitony@126.com; 2 Tianjin Key Laboratory of Urban Transport Emission Research & State Environmental Protection Key Laboratory of Urban Ambient Air Particulate Matter Pollution Prevention and Control, College of Environmental Science and Engineering, Nankai University, Tianjin 300071, China; liuyanwork@nankai.edu.cn; 3 Tianjin Youmei Environment Technology, Ltd., Tianjin 300300, China; yiying5120@126.com

**Keywords:** cadmium, tumor, colon cancer, reactive oxygen species, Wnt/β-catenin pathway

## Abstract

Background: Inhalation exposure to carcinogenic metals such as cadmium (Cd) is a significant global health concern linked to various cancers. However, the precise carcinogenic mechanism underlying inhalation exposure remains elusive. Methods: In this study, CT26 mouse colon cancer (CC) cells were implanted into BALB/c mice to establish CC mouse models. Some of the CC mice were implanted with intestinal stents. The mice were exposed to atomized oxygen and nitrogen (O_2_/N_2_) gas containing Cd. Results: Atmospheric Cd intensified inflammation in CC cells and heightened Nicotinamide Adenine Dinucleotide Phosphate (NADPH) Oxidase 1 (NOX1) activity, which is an indirect measurement of increased reactive oxygen species (ROS) production. This escalated ROS production triggered abnormal Wnt protein secretion, activated the Wnt/β-catenin signaling pathway, and stimulated CC cell proliferation. No discernible body weight effect was seen in the CC mice, possibly due to the later-stage tumor weight gain, which masked the changes in body weight. Cd facilitated colon tumor restructuring and cell migration at the later stage. The implantation of intestinal stents inhibited the expression of Superoxide Dismutase 1 (SOD1) in the colon tumors of the CC mice, with no evident effects on the expression levels of NOX1, SOD2, and Catalase (CAT) enzymes. Elevated ROS levels, indirectly reflected by enzyme activity, did not substantially impact the Wnt/β-catenin signaling pathway and even contributed to slowing its imbalance. Stent implantation eased the inflammation occurring in colon tumors by reducing CC cell proliferation but it induced discomfort in the mice, leading to a reduction in food intake and weight. Conclusions: Cd partially fosters CC tumorigenesis via the ROS-mediated Wnt/β-catenin signaling pathway. The effect of Cd on the invasive effect of intestinal stents in the cancerous colon is not significant.

## 1. Introduction

In recent years, attention has increasingly turned towards the issue of air pollution, which is paralleled by a growing body of research into the impact of air pollution on colon cancer (CC). CC stands as the second leading cause of cancer-related deaths globally [1]. The risk factors linked to CC are primarily categorized into modifiable elements (such as smoking, obesity, and diet) and non-modifiable factors (like family history, age, and sex). Recent studies have illustrated that exposure to environmental pollutants, notably heavy metals, could elevate the risk of CC. Among some common heavy metal pollutants are cadmium, arsenic, lead, mercury, and chromium [2,3,4].

Cadmium (Cd), a hazardous metal that is prevalent in the environment and commonly used in industrial processes, is classified as a human carcinogen by the International Agency for Research on Cancer (IARC) [5,6]. Mounting evidence indicates that the presence of Cd in aquatic environments can induce various adverse health outcomes in humans, including cancers [7,8], cardiovascular diseases [9,10], and inflammatory conditions [11,12]. Nevertheless, the impact of Cd in the atmosphere on human health, particularly concerning CC, remains elusive. Recent investigations have revealed a notable increase in plasma Cd levels among CC patients compared to healthy individuals [13]. Additionally, studies have demonstrated that Cd can prompt cellular transformation and carcinogenesis in human colon cell line CRL-1807 models [14]. There are speculations that these cellular responses are correlated with specific molecular mechanisms triggered by Cd exposure, such as the induction of oxidative stress, β-catenin mutation, the upregulation of cyclooxygenase-2 (COX-2), and the initiation of proinflammatory responses. However, further research is needed to comprehensively elucidate the intricate connections between Cd exposure and the molecular pathways involved in CC development. While the molecular mechanisms behind Cd-induced carcinogenesis necessitate further investigation, there is a consensus that Reactive Oxygen Species (ROS) play a pivotal role in cellular damage triggered by Cd, which subsequently culminates in cancer development. The Nicotinamide Adenine Dinucleotide Phosphate (NADPH) Oxidase (NOX) complex stands as a critical physiological system for ROS generation. Studies have revealed that Cd has the capability to activate NOX to produce ROS, which disrupts the integrity of the outer mitochondrial membrane, thereby engendering the production of superoxide radicals, hydroxyl radicals, and hydrogen peroxide, and thus causing cell damage [15]. In the context of CC cells, the Wnt/β-catenin pathway, which is controlled by ROS generated via NOX, regulates the proliferation of CC cells [16]. Wnt ligands constitute a diverse and highly conserved group of secreted proteins [17]. Their influence extends across various cellular processes, encompassing cell proliferation, stem cell self-renewal, the determination of cell fate, the establishment of cell polarity, and the orchestration of convergent extension behavior during cell migration [18,19,20]. Each of these orchestrated consequences, steered by Wnt signaling, is crucial for maintaining normal intestinal development. This pathway often exhibits differential regulation between normal and cancerous tissues, particularly in cancers such as hepatocellular carcinoma, prostate cancer, and CC [21]. Within healthy cells (Figure S1), in the absence of Wnt signaling, β-catenin is bound and regulated by a multi-subunit complex consisting of recombinant axis inhibition protein (Axin), Adenomatous Polyposis Coli (APC), Casein Kinase 1α (CK1α), and Glycogen Synthase Kinase 3β (GSK3β). This complex promotes β-catenin phosphorylation, facilitating its interaction with β-Transducin Repeat-Containing Proteins (β-TRCPs) and leading to subsequent ubiquitination and degradation, thus maintaining low β-catenin expression levels. Conversely, in cancer cells where Wnt signaling is active (Figure S2), Wnt signaling engages specific receptors like frizzled proteins, leading to low-density Lipoprotein Receptor-related Protein (LRP) phosphorylation. This culminates in the formation of the Wnt–Frizzled-LRP complex, triggering disheveled (DVL) activation and conglomerate aggregation toward the receptor. DVL activation intensifies GSK3β phosphorylation, thereby impeding β-catenin degradation [22]. Consequently, β-catenin accumulates in the nucleus, interacts with coactivators like T-Cell Factor and Lymphoid Enhancer Factor (TCF/LEF), and stimulates downstream Wnt target gene transcription, thus fostering cancer cell proliferation. Additionally, heightened ROS production incites an inflammatory cascade. The COX-2 pathway emerges as a pivotal inflammatory pathway implicated in CC [23]. Substantial data highlight the significantly elevated COX-2 gene expression in cancerous colon mucosa compared to healthy tissues [24,25,26]. The primary contributor to the carcinogenic effect of COX-2 is believed to be its chief metabolite, prostaglandin E2 (PGE2), which exerts its biological function by binding to its target receptor, prostaglandin receptor 4 (EP4) [27]. In addition, Iba1 is a macrophage/microglia-specific calcium-binding protein whose level can also reflect the degree of cellular inflammatory response [28]. However, the precise molecular mechanism by which Cd in the atmospheric milieu induces CC remains an area to be fully elucidated.

Advancements in medical devices and technologies have led to the gradual adoption of intestinal stents as the primary treatment for colon tract decompression. Evidence-based medicine has validated the short-term safety and efficacy of this approach [29,30,31]. Nevertheless, significant concerns persist regarding long-term tumor prognosis in CC patients who have undergone bowel stenting as a form of conversion therapy. Studies have indicated that the preoperative placement of bowel stents in CC patients disrupts tumor prognosis and potentially triggers tumor cell dissemination during the stent insertion process [32]. Further studies are, therefore, needed to verify the effect of intestinal stents on colon tumors. By studying the impact of intestinal stents on the CC pathology and Matrix Metalloproteinase-2 (MMP-2), MMP-9, and 8-hydroxy-2′-deoxyguanosine (8-OHdG) expression levels, the impact of intestinal stents on long-term tumor prognosis will be explained from a molecular perspective. Among these enzymes, MMP-2 primarily participates in tissue remodeling, cellular migration, and angiogenesis [33]. MMP-9 primarily participates in the activation and migration of inflammatory cells [34]. 8-OHdG serves as a commonly utilized biomarker to detect oxidative DNA damage resulting from oxidative stress [35]. However, the impact of atmospheric Cd on the invasiveness of stents in the cancerous colon remains largely unexplored.

**Figure 1:**
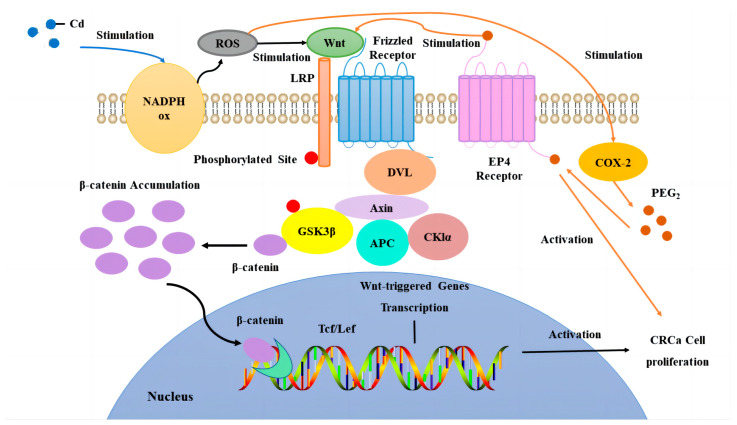
Hypotheses for the mechanism of Cd-induced proliferation of CC cells in atmospheric environments.

Hence, this study employed CT26WT CC mouse models with implanted stents. These mouse models were subjected to atomized air containing Cd via inhalation to mimic atmospheric exposure, aiming to investigate the influence of Cd in the environment on CC patients with implanted intestinal stents. The hypothesis posited here is that Cd in the atmosphere fosters the proliferation and dissemination of CC cells through a mechanism reliant on the NOX-ROS-COX-2-Wnt/β-catenin pathways. A preliminary verification of this hypothesis is illustrated in Figure 1.

## 2. Materials and Methods

### 2.1. Animal and Experimental Design

CT26 mouse CC cells, along with the culture medium, were procured from Wuhan Pricella Life Technology Co., Ltd. (Wuhan, Hubei Province, China) These CC cells were nurtured in 1640 complete medium containing 10% Fetal Bovine Serum (FBS) and 1% bis-antibodies in a 5% carbon dioxide (CO_2_) incubator at 37 °C. Forty-five 6-week-old female BALB/c mice were acquired from Beijing Sipeifu Biotechnology Co., Ltd. (Beijing, China) and were housed in accordance with the institutional animal care guidelines. To facilitate acclimation, the mice were allowed a week in the new environment before utilization. The mice were housed in plastic cages (5 mice/cage) under the following conditions: a relative humidity of 50 ± 10%, a 12/12 h light/dark cycle, and a temperature of 23 ± 2 °C.

For the implantation of CC cells, 1 × 10^7^/mL of cells (200 μL/piece) was cultured on the mucosa of the posterior colon wall in the mice, and tumor formation was observed over a 2-week period utilizing a small-animal live imaging device (InVivo Smart-LF, VISQUE, Seoul, Republic of Korea). The BALB/c mice were then randomly divided into 9 groups based on body weight; the healthy groups included a blank group (H-Blank), a control group (H-Control), and a Cd group (H-Cd). The groups with CC cells but without an intestinal stent included a blank group (C-Blank), a control group (C-Control), and a Cd group (C-Cd). The groups with CC cells and an intestinal stent included a blank group (C-S-Blank), a control group (C-S-Control), and a Cd group (C-S-Cd). The mice in the intestinal stent groups were implanted with metal spring coils (length of 5 mm, inner diameter of 2 mm, outer diameter of 3 mm, and wire diameter of 0.2 mm) through the anus and cultured for 2 weeks. The Cd groups were exposed to a simulated environment of oxygen and nitrogen (O_2_/N_2_) mixed with atomized gas containing Cd for the subsequent 4 weeks. The blank groups were exposed to the actual environment without gas introduction, while the control groups were exposed to a simulated environment with the introduction of O_2_/N_2_ mixed with atomized gas. Throughout the experiment, the mice were weighed twice weekly, and any signs of weight loss were closely monitored. At 27 weeks of age, all mice were euthanized using CO_2_ asphyxiation, and whole-colon intestinal tissues were collected. The tissue samples were promptly fixed in 10% formalin or frozen in liquid nitrogen for further analysis. 

A corresponding preliminary experiment was conducted before the formal experiment in this study. The experimental design was tested for validity and efficacy. Groups of 5 mice were verified to have ≥80% power on the primary endpoint, and it was confirmed that the number of rats in each group was the minimum necessary to ensure that the experiment was able to detect the intended effect. All experiments were conducted in accordance with the biomedical research guidelines stipulated by the Experimental Biology Society of Tianjin People’s Hospital and were approved by the Animal Use Experimental Ethics Committee of Tianjin Union Medical Center.

### 2.2. Establishment of the Simulation Environment

The reference was drawn from the average concentration of Cd in the atmospheric environment spanning from January 2016 to February 2017, specifically under heavy pollution weather conditions (AQI > 200), in Heping District, Tianjin. During this period, the average concentration was recorded at 4.33 ng/m^3^. As demonstrated in Formula (1), the estimated quantity of Cd inhaled by humans in such an atmospheric environment amounted to 46.76 ng per day, whereas the estimated intake for BALB/c mice in this atmospheric environment was 0.13 ng per day. The formula is as follows:

(1) E=C×B×t

where *E* is the respiratory exposure to Cd in the atmospheric environment, in ng; *C* is the average concentration of Cd in the atmospheric environment, in ng/m^3^; and *B* is the respiratory rate, in m^3^/d. The respiratory rate of BALB/c mice is 19.16 mL/min [36], which is 0.03 m^3^/d, and the respiratory rate of humans is 10.8 m^3^/d [37]; *t* is the exposure time, in d. The Cd solution used in the experiment to simulate a polluted atmospheric environment was a solution derived from the Cd inductively coupled plasma (ICP) standard solution obtained from O2si, USA. To align with the inhalation volume for both humans and BALB/c mice, the Cd ICP standard solution was prepared at a concentration of 0.23 μg/mL. The compressed air inlet of the medical nebulizer was connected to a fresh air cylinder (21% O_2_/79% N_2_) to generate dispersion nebulization gas. The actual concentration of cadmium in the atomized environment was 4.1~4.5 ng/m^3^.

### 2.3. In Vivo Imaging

The mice were anesthetized with gas. The mice were intraperitoneally injected with D-luciferin sodium salt physiological saline solution (10 mg/mL) at a dose of 150 mg/kg. Fifteen minutes after injection, the anesthetized mice were placed in an in vivo imaging device for observation and imaging.

### 2.4. Histopathology

All tumor and mucosa specimens underwent hematoxylin and eosin (H&E) staining for histopathological assessment. The samples were fixed with paraformaldehyde and then decalcified in a decalcifying solution. The dehydration of the samples was performed by using alcohol, and the removal of the tissues from the paraffin was carried out using a xylene solution. The tissue samples underwent embedding by being dipped in a melted wax solution and placed in an embedding frame. After being positioned appropriately according to the embedding surface requirements, the samples were cooled on a −20 °C freezing platform until the wax solidified. After solidification, the wax block was removed from the embedding frame, trimmed, and placed on a paraffin microtome for slicing. The resulting slices were floated on 40 °C warm water in a spreading machine to flatten the tissue. Subsequently, the tissue was picked up with a glass slide and placed in a 60 °C oven for baking. Following baking, the slices were dewaxed in xylene twice and dehydrated using absolute ethanol. Hydration of the sample sections was performed using a series of ethanol concentrations (95%, 80%, and 70%) and distilled water. Subsequent staining involved hematoxylin staining solution application, followed by differentiation. The samples underwent dehydration, transparency treatment, and sealing. Observation and imaging were conducted using a fluorescence microscope (ECLIPSE Ci, Nikon, Tokyo, Japan) to analyze the stained tissue sections.

### 2.5. Immunohistochemistry

To achieve cell permeability and block endogenous peroxidase, the sample sections underwent immersion in a blocking/permeabilization solution (at room temperature and shielded from light). To uncover the antigen-determining area for antigen retrieval, the slices were submerged in 0.01 M sodium citrate buffer (pH = 6.0) and heated in a microwave oven until boiling, with the regular replenishment of the solution to prevent desiccation. To obstruct the influence of non-specific proteins, the surrounding tissue was outlined using a histochemical pen, followed by the application of 5% sheep serum within the outlined area. The diluted primary antibody was directly added and allowed to incubate overnight at 4 °C. Following this, the diluted secondary antibody was applied, and the sections were placed in a constant-temperature oven at 37 °C. The subsequent steps involved the addition of streptavidin peroxidase (SP) conjugates, placement in a 37 °C oven, and the introduction of diaminobenzidine (DAB). Staining progression was observed under a fluorescence microscope while controlling the duration based on the color observed. Subsequently, a hematoxylin staining solution was applied to stain nuclear proteins, followed by the use of phosphate-buffered saline (PBS) to restore their blue coloration. The samples underwent dehydration, transparency treatment, and sealing. Observation and imaging were conducted using a fluorescence microscope to analyze the stained tissue sections.

### 2.6. Western Blot Analysis

Western blot analysis was conducted utilizing specific antibodies targeting β-catenin (1:1000, BIOSS), phospho-GSK3β (1:1000, BIOSS), actin (1:3000, Affinity), SOD1 (1:1000, Affinity), SOD2 (1:1000, Affinity), CAT (1:1000, BIOSS), and NOX1 (1:1000, Affinity).

### 2.7. Statistical Analysis

Differences between treatment groups were assessed through analysis of variance (ANOVA). Statistical significance was determined at *p* < 0.05. For cases where significant differences were observed, specific post hoc comparisons between treatment groups were conducted using the Student–Newman–Keuls test. All statistical analyses were conducted using SPSS software (version 27.0, SPSS Inc., Chicago, IL, USA). The data were assessed for normality and homoskedasticity before performing the ANOVA (analysis of variance) and the Student–Newman–Keuls test. Normality was tested using the Shapiro–Wilk test, and the results showed that the data conformed to a normal distribution (*p* > 0.05). Homoscedasticity was assessed using Levene’s test, and the results showed that each group of data had similar variance (*p* > 0.05). These results showed that the data of this study met the basic assumptions of ANOVA and the Student–Newman–Keuls test, thus providing a reliable basis for subsequent statistical analysis.

## 3. Results

### 3.1. Colon Tumor Invasion and Metastasis Capabilities

**Figure 2:**
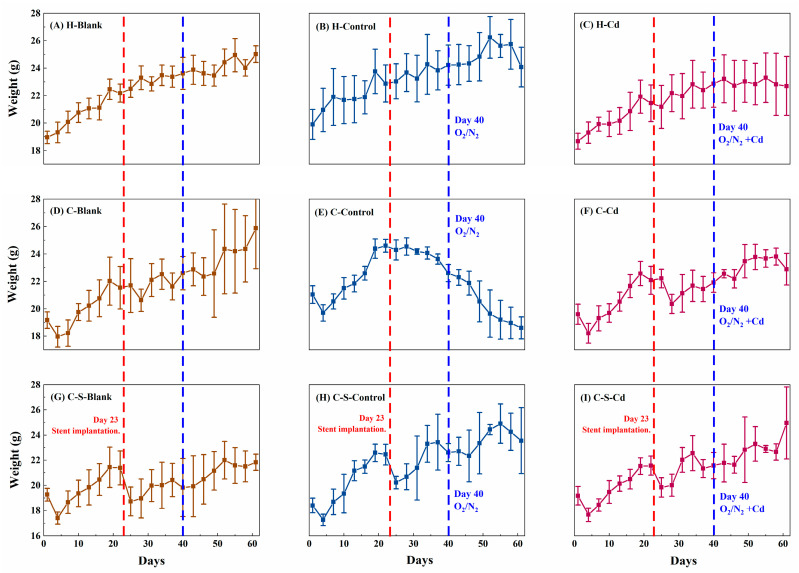
Changes in body weight of mice (g). Data were means ± SEM.

The mice lost weight within 4 days after the CC cells were implanted, as shown in Figure 2D,I. With tumor progression, a gradual adaptation was apparent. However, upon the implantation of intestinal stents on day 23, the mice with CC cells experienced weight loss, as indicated in Figure 2G,I. Interestingly, the injection of O_2_/N_2_ atomized gas and Cd-O_2_/N_2_ atomized gas exhibited no notable impact on the mice’s body weight compared to the control groups.

**Figure 3:**
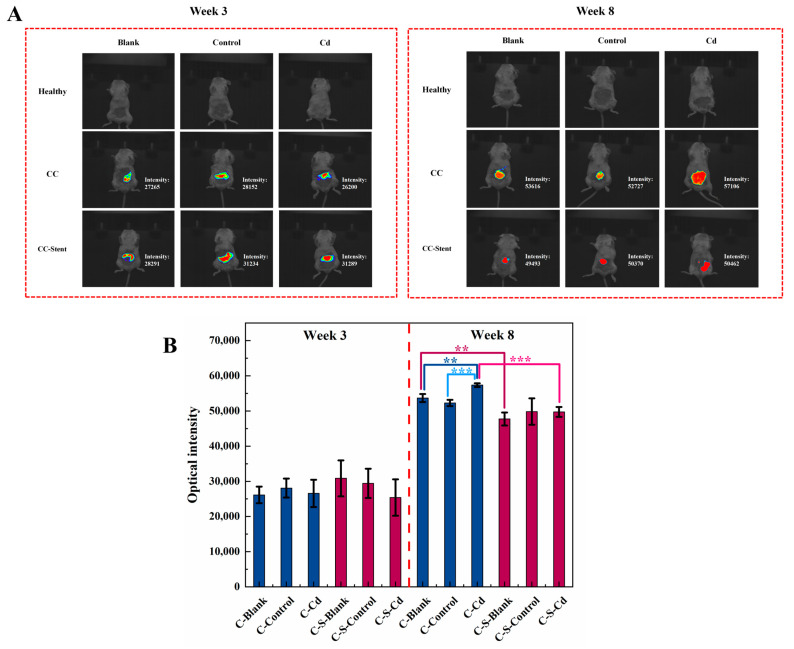
In vivo imaging was performed at the 3rd and 8th weeks after the start of the experiment (before sampling) to observe the (**A**) in situ tumor formation of the colon and (**B**) the changes in fluorescence intensity. (**B**) Data were means ± SEM of 5 mice. ** *p* < 0.01, *** *p* < 0.001. Among them, if there is no asterisk between the two columns, it means there is no statistical significance.

As illustrated in Figure 3, there was extensive proliferation of the CC cells from the third to the eighth week, resulting in an enhancement of fluorescence signals within the colon tumor tissues. Notably, compared to the blank and control groups, the injection of Cd-O_2_/N_2_ atomized gas further significantly intensified the proliferation of CC cells (C-Blank: *p* < 0.01; C-Control: *p* < 0.001). However, upon the implantation of intestinal stents, the proliferative capacity of CC cells showed a statistically significant decrease (C-Blank: *p* < 0.01; C-Cd: *p* < 0.001). 

**Figure 4:**
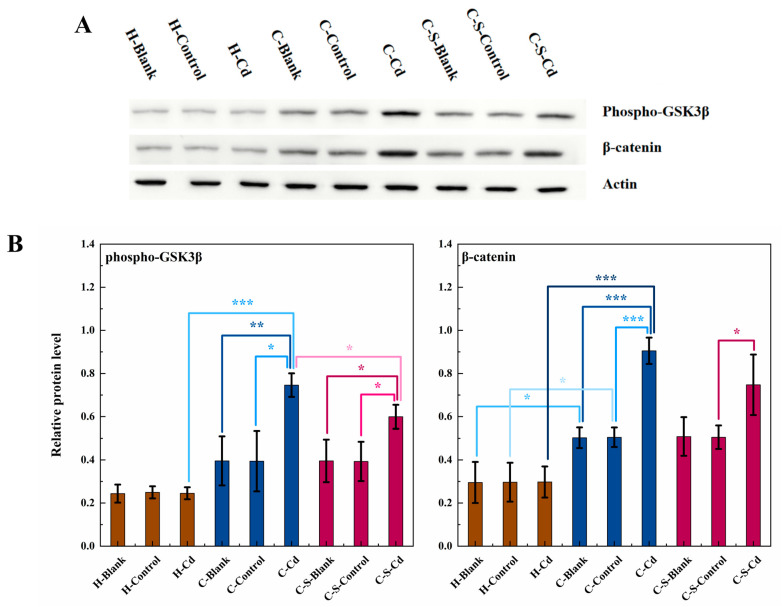
The expression of phospho-GSK3β and β-catenin in colon tumors of mice in each group was detected. (**A**) Expression of phospho-GSK3β and β-catenin in colon tumors was determined by immunoblotting. Expression of actin served as an internal control. (**B**) Relative levels of β-catenin and phospho-GSK3β in colon tumors. Data were means ± SEM of 5 mice. * *p* < 0.05, ** *p* < 0.01, *** *p* < 0.001. Among them, if there is no asterisk between the two columns, it means there is no statistical significance.

In comparison to the healthy mice, statistically significantly elevated expression levels were observed for β-catenin (H-Blank and H-Control: *p* < 0.05; H-Cd: *p* < 0.001) and phospho-GSK3β (H-Cd: *p* < 0.001) within the colon tumors of the CC mice, as depicted in Figure 4. The injection of Cd-O_2_/N_2_ atomized gas further significantly augmented the expression levels of β-catenin (C-Blank and C-Control: *p* < 0.001; C-S-Control: *p* < 0.05) and phospho-GSK3β (C-Blank: *p* < 0.01; C-Control, C-S-Blank and C-S-Control: *p* < 0.05). Conversely, the implantation of intestinal stents statistically significantly suppressed the expression of phospho-GSK3β (C-Cd: *p* < 0.05). However, its effect on the expression level of β-catenin was not significant. In addition, the implantation of intestinal stents had no statistically significant effect on the expression levels of β-catenin and phospho-GSK3β in the colon tumors of the CC mice in the blank and control groups.

**Figure 5:**
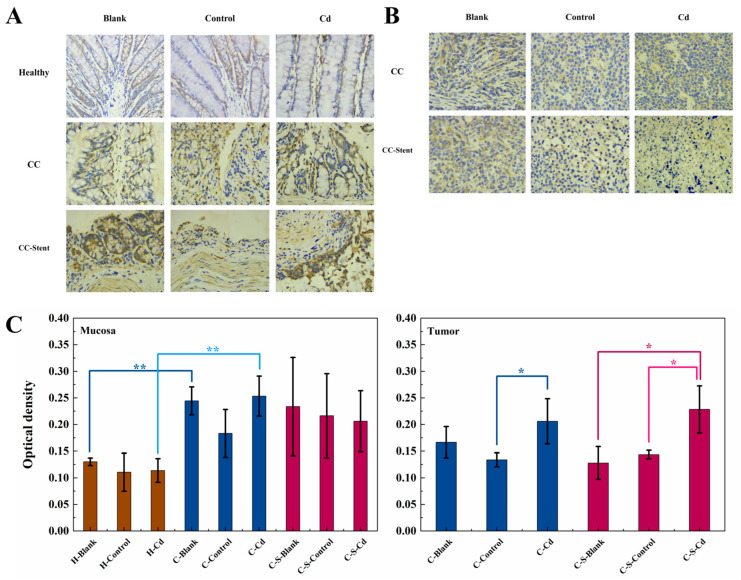
The expression of MMP-2 in colon tumors and mucosa of mice in each group was detected. (**A**) Immunohistochemical staining of MMP-2 in colonic mucosa in indicated treatments. Magnification was 400×. (**B**) Immunohistochemical staining of MMP-2 in colonic tumors in indicated treatments. Magnification was 400×. (**C**) Optical density was used to represent the expression of MMP-2 in colon mucosa and tumors. Data were means ± SEM of 5 mice. * *p* < 0.05, ** *p* < 0.01. Among them, if there is no asterisk between the two columns, it means there is no statistical significance.

The assessment of colon tumor invasion and metastatic potential involved the examination and analysis of MMP-2 expression. In comparison to the healthy mice, statistically significantly heightened MMP-2 expression was observed in the colon mucosa of the CC mice (H-Blank and H-Cd: *p* < 0.01), as depicted in Figure 5. Notably, the injection of Cd-O_2_/N_2_ atomized gas statistically significantly amplified MMP-2 expression in the colon tumors of the CC mice relative to the blank and control groups (C-Control, C-S-Blank, and C-S-Control: *p* < 0.05). The impact of intestinal stent implantation on MMP-2 expression level was not statistically significant.

### 3.2. Oxidative Stress

**Figure 6:**
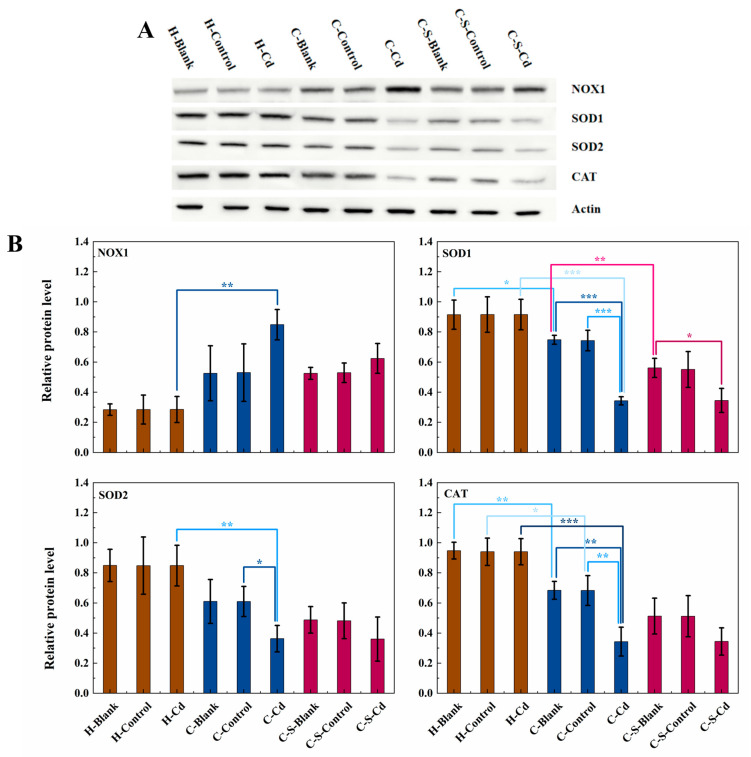
The expression of NOX1, SOD1, SOD2, and CAT in colon tumors of mice in each group was detected. (**A**) Expression of NOX1, SOD1, SOD2, and CAT in colon tumors was determined by immunoblotting. Expression of actin served as an internal control. (**B**) Relative levels of NOX1, SOD1, SOD2, and CAT in colon tumors. Data are means ± SEM of 5 mice. * *p* < 0.05, ** *p* < 0.01, *** *p* < 0.001. Among them, if there is no asterisk between the two columns, it means there is no statistical significance.

Carcinogenic metals are renowned for their capacity to induce ROS [38]. Due to resource limitations that hindered the ability to conduct direct ROS measurements, this study instead measured the activities of NOX1, SOD1, SOD2, and CAT enzymes, which are key players in the cellular anti-oxidant defense system and in oxidative stress, to indirectly illustrate changes in the production of ROS [39,40,41]. As depicted in Figure 6, NOX1 expression levels were statistically significantly increased in the colon tumors of the CC mice when compared to their healthy counterparts (H-Cd: *p* < 0.05). Notably, the alterations in the expression levels of NOX1, β-catenin, and phospho-GSK3β followed a similar pattern. Additionally, statistically significantly decreased expression levels were observed for SOD1 (H-Blank: *p* < 0.05; H-Cd: *p* < 0.001), SOD2 (H-Cd: *p* < 0.01), and CAT (H-Blank: *p* < 0.01; H-Control and H-Cd: *p* < 0.001) in these tumors. The effect on the expression levels of NOX1 and SOD2 after the transplantation of CC cells into the mice in the blank and control groups was not statistically significant. The injection of Cd-O_2_/N_2_ atomized gas further significantly suppressed the expression of SOD1 (C-Blank and C-Control: *p* < 0.001; C-S-Blank: *p* < 0.05), SOD2 (C-Control: *p* < 0.05), and CAT (C-Blank and C-Control: *p* < 0.01) in the colon tumors of the CC mice. The effect of Cd-O_2_/N_2_ atomized gas on the expression levels of NOX1, SOD2, and CAT in the colon tumors of the CC mice with intestinal stents implanted in them was not statistically significant. Conversely, the implantation of intestinal stents statistically significantly inhibited SOD1 expression in the colon tumors of the CC mice (C-Blank: *p* < 0.01). However, the effect on the expression of NOX1, SOD2, and CAT was not statistically significant. In addition, the implantation of intestinal stents had no statistically significant effect on the expression of NOX1, SOD1, SOD2, and CAT in the colon tumors of the CC mice exposed to a Cd-O_2_/N_2_ atomized gas environment.

**Figure 7:**
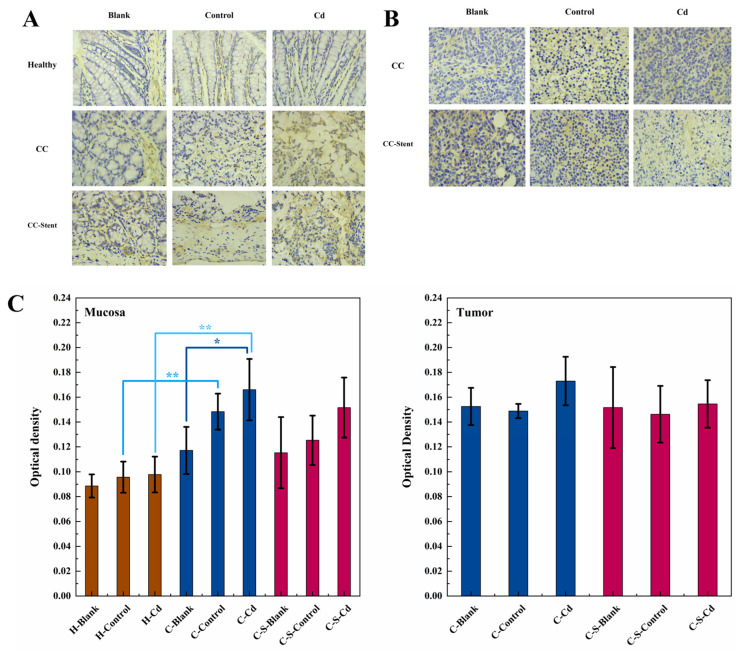
The expression of 8-OHdG in colon tumor and mucosa of mice in each group was detected. (**A**) Immunohistochemical staining of 8-OHdG in colonic mucosa in indicated treatments. Magnification was 100×. (**B**) Immunohistochemical staining of 8-OHdG in colonic tumor in indicated treatments. Magnification was 100×. (**C**) Optical density was used to represent the expression of 8-OHdG in colon mucosa and tumor. Data are means ± SEM of 5 mice. * *p* < 0.05, ** *p* < 0.01. Among them, if there is no asterisk between the two columns, it means there is no statistical significance.

Relative to the healthy mice, statistically significantly elevated expression levels of 8-OHdG were observed within the colon mucosa of the CC-afflicted mice (H-Control and H-Cd: *p* < 0.01), as illustrated in Figure 7. The injection of Cd-O_2_/N_2_ atomized gas further significantly amplified the expression of 8-OHdG in the colon mucosa (C-Blank: *p* < 0.05). The implantation of intestinal stents had no significant effect on the expression of 8-OHdG in the colon mucosa of the CC mice.

### 3.3. Inflammation

**Figure 8:**
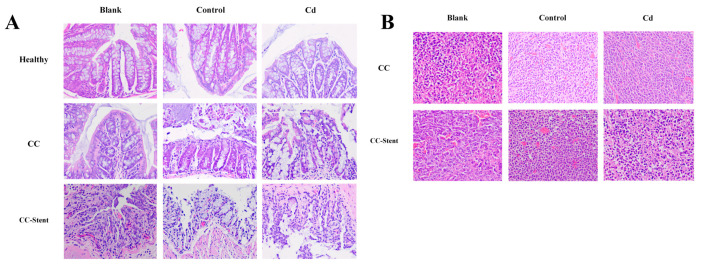
The pathology of colon tumor and mucosa of mice in each group was observed. (**A**) Representative H&E staining demonstrating mucosa from indicated treatments (red arrow indicates inflammatory cell infiltration). Magnification was 100×. (**B**) Representative H&E staining demonstrating tumors from indicated treatments. Magnification was 100×.

As depicted in Figure 8, the colon mucosa of the healthy mice exhibited a well-maintained structure characterized by neatly arranged glands and an absence of inflammatory cell infiltration. Conversely, the colon mucosa of the CC mice displayed evident damage, with disordered glandular arrangement and noticeable infiltration of inflammatory cells. Remarkably, the mucosa of the CC mice implanted with intestinal stents exhibited severe damage, featuring disorganized glands and pronounced inflammatory cell infiltration. Notably, the tumors observed across in all groups of mice exhibited irregular nuclear morphology and variable sizes. Cell arrangement appeared disordered, lacked a distinct tissue structure, and showcased irregular intercellular spaces. 

**Figure 9:**
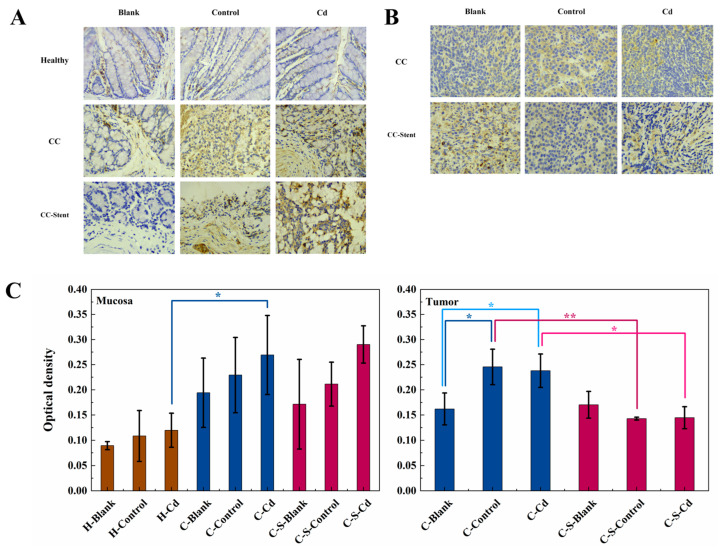
The expression of MMP-9 in colon tumors and mucosa of mice in each group was detected. (**A**) Immunohistochemical staining of MMP-9 in colonic mucosa in indicated treatments. Magnification was 400×. (**B**) Immunohistochemical staining of MMP-9 in colonic tumors in indicated treatments. Magnification was 400×. (**C**) Optical density was used to represent the expression of MMP-9 in colon mucosa and tumors. Data are means ± SEM of 5 mice. * *p* < 0.05, ** *p* < 0.01. If there is no asterisk between the two columns, it means there is no statistical significance.

Relative to the healthy mice, statistically significantly heightened MMP-9 expression was observed within the colon mucosa of the CC mice (H-Cd: *p* < 0.05), as depicted in Figure 9. The implantation of CC cells had no statistically significant effect on the expression levels of MMP-9 in the colon mucosa of the CC mice in the blank and control groups. The injection of Cd-O_2_/N_2_ atomized gas statistically significantly suppressed the expression of MMP-9 in the colon tumors of the CC mice (C-Blank: *p* < 0.05). However, the effect on MMP-9 expression in the colon mucosa of the CC mice that had been implanted with intestinal stents was not significant. Similarly, the implantation of intestinal stents statistically significantly suppressed MMP-9 expression in the colon tumors of the CC mice (C-Control: *p* < 0.01; C-Cd: *p* < 0.05). Variations in MMP-9 expression were noted in the colon tumors of the CC mice in the blank and control groups (*p* < 0.05). 

**Figure 10:**
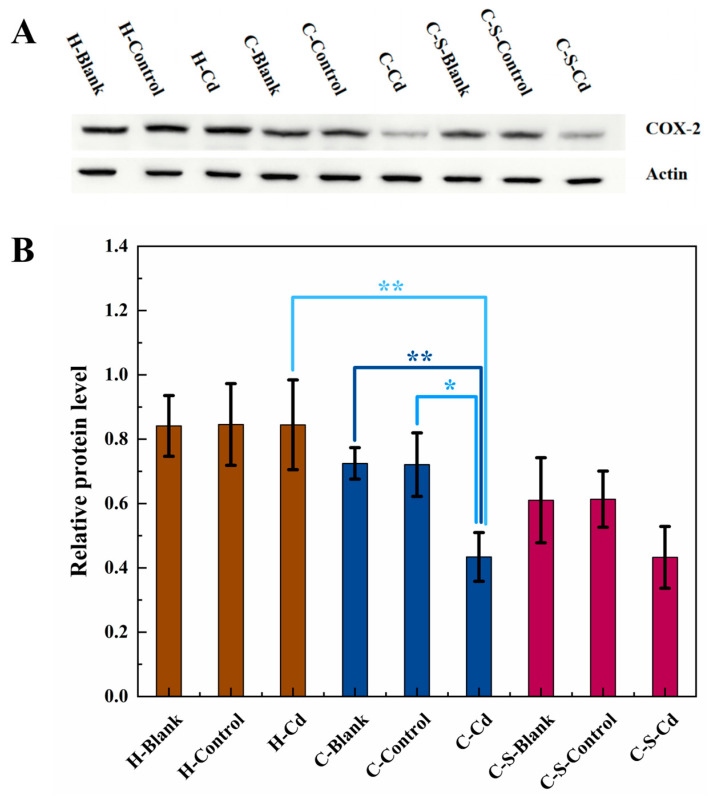
The expression of COX-2 in colon tumor of mice in each group was detected. (**A**) Expression of COX-2 in colon tumors was determined by immunoblotting. Expression of actin served as an internal control. (**B**) Relative levels of COX-2 in colon tumors. Data are means ± SEM of 5 mice. * *p* < 0.05, ** *p* < 0.01. If there is no asterisk between the two columns, it means there is no statistical significance.

As depicted in Figure 10, the expression of COX-2 was notably suppressed in the colon tumors of the CC mice compared to their healthy counterparts (H-Cd: *p* < 0.01). The implantation of CC cells had no statistically significant effect on the expression levels of COX-2 in the colon mucosa of the CC mice in the blank and control groups. The injection of Cd-O_2_/N_2_ atomized gas further significantly inhibited COX-2 expression within these colon tumors (C-Blank: *p* < 0.01; C-Control: *p* < 0.05), but the effect on COX-2 expression in the colon mucosa of the CC mice that had been implanted with intestinal stents was not significant.

**Figure 11:**
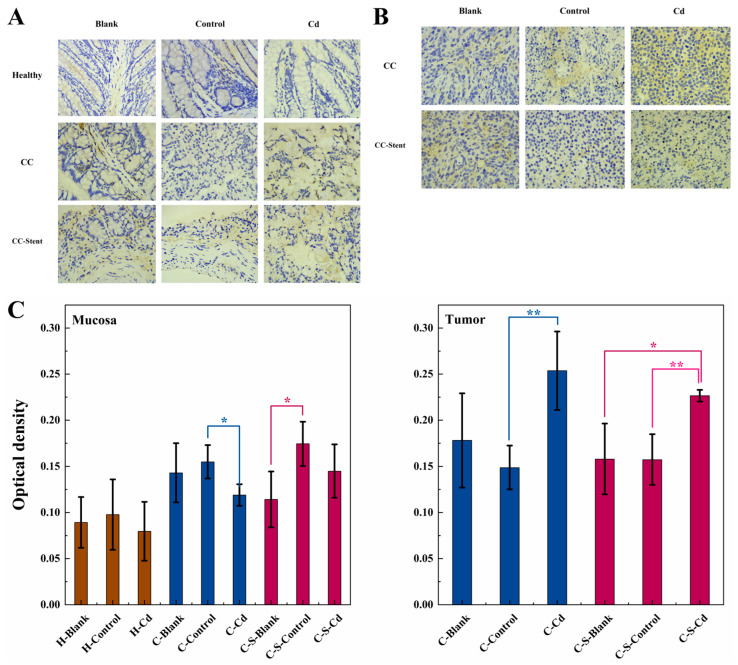
The expression of Iba1 in colon tumors and mucosa of mice in each group was detected. (**A**) Immunohistochemical staining of Iba1 in colonic mucosa in indicated treatments. Magnification was 400×. (**B**) Immunohistochemical staining of Iba1 in colonic tumors in indicated treatments. Magnification was 400×. (**C**) Optical density was used to represent the expression of Iba1 in colon mucosa and tumors. Data are means ± SEM of 5 mice. * *p* < 0.05, ** *p* < 0.01. If there is no asterisk between the two columns, it means there is no statistical significance among them.

The implantation of CC cells had no significant effect on Iba1 expression. Notably, the injection of Cd-O_2_/N_2_ atomized gas led to a statistically significant reduction in Iba1 expression in the colon mucosa (C-Control: *p* < 0.05) and tumors (C-Control: *p* < 0.01; C-S-Blank: *p* < 0.05; C-S-Control: *p* < 0.01), as depicted in Figure 11. Significant variation in the expression of Iba1 was observed in the colon mucosa of the blank and control groups following stent implantation. 

## 4. Discussion

In this study, mouse models of CC were employed to investigate the implications of exposure to the heavy metal Cd in the atmospheric environment on CC progression and its impact on the invasiveness of intestinal stents within the cancerous colon. An abnormal secretion of Wnt protein within CC cells triggered an unconventional activation of the Wnt/β-catenin signaling pathway, culminating in the phosphorylation of GSK3β. Consequently, this cascade hindered the ubiquitination process of β-catenin, resulting in its intracellular accumulation and the subsequent promotion of CC cell proliferation [42]. The proliferation demands imposed on CC cells led to increased energy consumption, contributing to an initial decline in body weight among the CC mice [43]. Subsequently, in the later stages of the trial, an observable rise in tissue protein degradation within the colon mucosa of the CC mice significantly contributed to the invasive nature of colon tumors. Additionally, Cd amplified CC cell proliferation by further stimulating GSK3β phosphorylation and leading to subsequent intracellular β-catenin accumulation. This finding aligns with the observations made by Wei [44], who noted the role of Cd in advancing malignant tumors by inhibiting GSK3β activity while concurrently enhancing β-catenin expression. Similarly, Chakraborty [45] suggested that Cd-induced alterations in cancerous conditions plausibly occur through the Wnt signaling pathways. This enhancement did not produce statistically significant changes in the body weight of the CC mice compared to the healthy mice, likely due to the overshadowing effect of increased tumor tissue weight in the later stages of the experiment. Additionally, in the latter phase of the study, Cd notably facilitated colon tumor remodeling and instigated tumor cell migration. This corroborates with Wang’s [46] findings, wherein Cd exposure induced MMP-2 mRNA expression and consequently fostered the migration and invasion capabilities of human breast cancer cells. The implantation of intestinal stents alleviated GSK3β phosphorylation and reduced intracellular β-catenin accumulation, thereby slowing down CC cell proliferation. A stent takes up part of the intestinal space and prevents colon tumors from expanding. Additionally, intestinal stents can reduce the blood supply to colon tumors. Colon tumor growth requires large amounts of oxygen and nutrients, and if the blood supply is reduced, the reproductive capacity of CC cells will be inhibited [47]. This aligns with Matsuda’s [48] observations, suggesting that the mechanical compression induced by intestinal stents could hinder cancer cell proliferation in cases of malignant large bowel obstruction. However, the implantation of intestinal stents induced gastrointestinal discomfort in the CC mice, resulting in reduced food intake and weight loss. Furthermore, their implantation easily triggered inflammation and infection, compelling the body to expend additional energy to manage the immune response [49].

ROS generated by oxidative stress in the colon mucosa of the CC mice, indirectly reflected by enzyme activity, induced oxidative DNA damage, significantly contributing to the proliferation and spread of CC cells. This aligns with the conclusions drawn by Nilsson [50]. Additionally, NOX1 activity was heightened within CC cells; this is an indirect indicator of increased ROS, and increased ROS levels abnormally activate the Wnt/β-catenin signaling pathway by disrupting Wnt protein secretion. Consequently, this abnormal activation fosters the uncontrolled proliferation of CC cells [51,52,53]. Cd exacerbates ROS production by further amplifying NOX1 activity in CC cells, a phenomenon also observed by Tyagi [54] and Lian [55]. The implantation of intestinal stents inhibited the expression of SOD1 in the colon tumors of the CC mice. However, the impact on NOX1, SOD2, and CAT enzyme activities was not evident. SOD, which is categorized into three groups (SOD1, SOD2, and SOD3), plays a crucial role in maintaining intracellular ROS homeostasis [56]. Specifically, SOD1 facilitates the dismutation of O^2−^ into H_2_O_2_, a stable ROS messenger that is pivotal for regulating oxidative stress and supporting oncogene-driven cancer cell proliferation [57]. SOD2 scavenges superoxide radicals formed in the respiratory electron transport chain, thereby impacting cell cycle signaling and cancer progression [58]. CAT acts as an enzymatic scavenger by catalyzing the breakdown of H_2_O_2_ into oxygen and water, shielding cells from H_2_O_2_ toxicity and serving as a key element in the biological defense system [59]. Consequently, it was indirectly demonstrated via measurements of enzyme activity that there was no substantial increase in intracellular ROS after intestinal stent implantation, suggesting a less pronounced impact on the Wnt/β-catenin signaling pathway. This effect even contributes to the slowing down of the dysregulation of the Wnt/β-catenin signaling pathway.

The CC mice exhibited colon mucosal damage, disordered glandular arrangement, and infiltration of the inflammatory cells. However, upon the implantation of intestinal stents, severe damage to the colon mucosa and a noticeable infiltration of inflammatory cells were observed. Cd exhibited a dual impact on the immune response within the colon mucosa of the CC mice. It inhibited the expression of Iba1, likely due to its induction of oxidative stress, thus triggering the generation of oxygen free radicals in colon cells. This unfavorable effect on immune cells resulted in the a reduced production of inflammatory cytokines, thereby mitigating the inflammatory response [60]. Conversely, Cd promoted the expression of Iba1 in the colon tumors of the CC mice. This indicated that Cd heightened the inflammatory response in CC cells, consequently fostering the proliferation of colon tumor cells. It stimulated the activity of certain immune cells, notably macrophages, thereby triggering inflammatory reactions and elevating the expression levels of immune markers such as Iba1. A similar study by Yang [61] also reported increased gliosis, as indicated by a rise in the number of Iba1-positive cells, following Cd poisoning. Moreover, intestinal stents inhibited the expression of MMP-9 and COX-2 in colon tumors. This inhibition is linked to the ability of intestinal stents to suppress the activity of inflammation-related cells, possibly including those responsible for producing MMP-9 and COX-2 in colon tumors. However, this speculation necessitates further validation and investigation. 

There are several points in this study that require further investigation. First, there was a difference in the expression of MMP-9 between the C-Blank and C-Control groups, but there was no difference between the C-S-Blank and C-S-Control groups. This might be due to the control group being exposed to O_2_/N_2_ atomized gas, which increased the proportion of oxygen and nitrogen in the atmospheric environment under which the mice lived, which, in turn, might lead to oxidative reactions in CC cells and promote the expression of MMP-9 in the tumors of the CC mice. The implantation of intestinal stents may inhibit this reaction. However, there is currently a lack of research in this area. Therefore, more research is needed to verify this speculation. Moreover, the expression of Iba1 was opposite to that of MMP-9. This difference might occur due to the reaction of the metal stent with substances in the actual atmospheric environment. Second, compared to the C-control and C-Cd mice, the mice in the C-S-Control and C-S-Cd groups exhibited significantly lower MMP-9 expression levels, but the mice in the C-S-Blank group showed a significantly lower expression level than the C-Blank mice. There was no significant change in the expression level of MMP-9 across the groups. This might be due to the difference in gas composition in the actual and simulated atmospheric environments. This study did not research the interaction of substances in the actual atmospheric environment and gas components with cadmium, intestinal stents, and colon cancer cells. Thus, further research is needed in the future.

## 5. Conclusions

The presence of the heavy metal Cd in an atmospheric milieu acts as a catalyst in the progression of tumorigenesis within murine models of CC. The implementation of intestinal stents demonstrates a mitigating effect on tumor incidence within these CC murine models. Significantly, an aberrant activation of the ROS-mediated Wnt/β-catenin signaling pathway emerges as a pivotal mechanism contributing to the facilitation of this tumorigenic promotion. Cd partially fosters CC tumorigenesis via the ROS-mediated Wnt/β-catenin signaling pathway. The effect of Cd on the invasive effect of intestinal stents in the cancerous colon is not significant.

## Data Availability

The original data presented in the study are included in the article/Supplementary Materials; further inquiries can be directed to the corresponding authors.

## Supplementary Materials

The following supporting information can be downloaded at: https://www.mdpi.com/article/10.3390/toxics12030215/s1, Figure S1: Basic conditions in healthy cells.; Figure S2: Cumulative mutations of β-catenin in cancer cells.
