# Supplementary material for: The Impact of Atmospheric Cadmium Exposure on Colon Cancer and the Invasiveness of Intestinal Stents in the Cancerous Colon
Source: Toxics. 2024 Mar 14;12(3):215. doi: 10.3390/toxics12030215 (PMC10975477; doi:10.3390/toxics12030215)
Supplement: Supplementary file 1 [file toxics-12-00215-s001.zip › toxics-2825945-supplementary.pdf]

Supplementary Material

The Impact of Atmospheric Cadmium Exposure on Colon Cancer and the Invasiveness of Intestinal Stents in the Cancerous Colon

Shuai Zhang [1], Ruikang Li [2,*], Jing Xu [1,*], Yan Liu [2] and Yanjie Zhang [3]

[1] Department of General Surgery, Tianjin Union Medical Center, No. 190 Jieyuan Road, Hongqiao District, Tianjin 300121, China; gelinbarlitony@126.com
[2] Tianjin Key Laboratory of Urban Transport Emission Research & State Environmental Protection Key Laboratory of Urban Ambient Air Particulate Matter Pollution Prevention and Control, College of Environmental Science and Engineering, Nankai University, Tianjin 300071, China; liuyanwork@nankai.edu.cn
[3] Tianjin Youmei Environment Technology, Ltd., Tianjin 300300, China; yiying5120@126.com
* Correspondence: nklrkstudy@163.com (R.L.); xujingdoc@126.com (J.X.)

The supplementary material includes two figures.

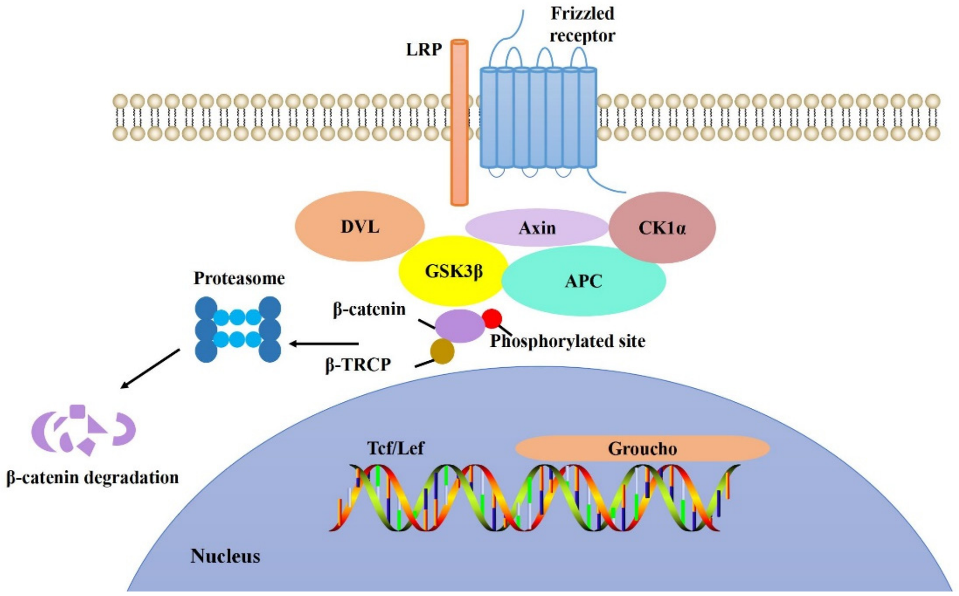

Figure S1. Basic conditions in healthy cells.

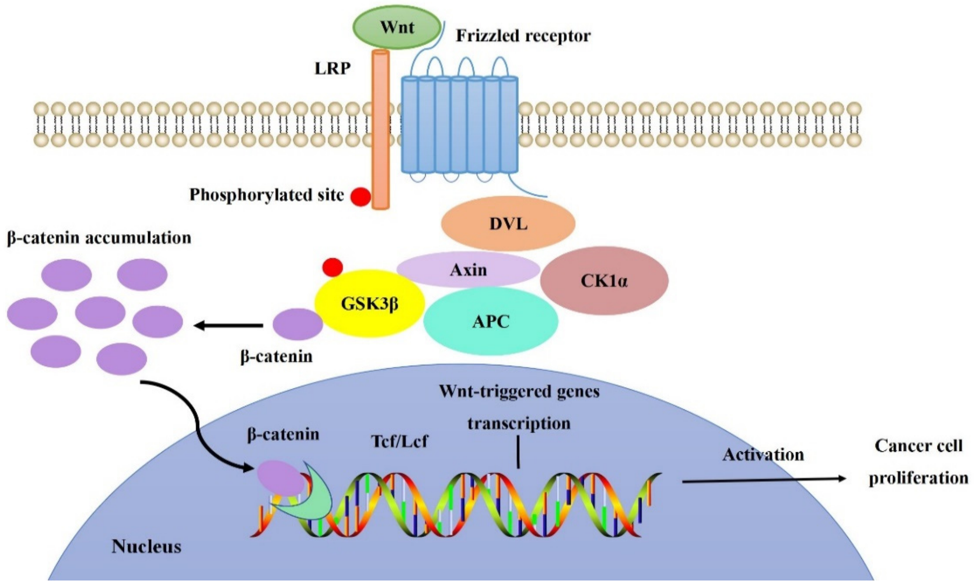

Figure S2. Cumulative mutations of β-catenin in cancer cells.
